# Anti-Cracking TEOS-Based Hybrid Materials as Reinforcement Agents for Paper Relics

**DOI:** 10.3390/molecules29081834

**Published:** 2024-04-17

**Authors:** Mengruo Wu, Le Mu, Zhiyue Zhang, Xiangna Han, Hong Guo, Liuyang Han

**Affiliations:** 1 Key Laboratory of Archaeomaterials and Conservation, Ministry of Education, Institute for Cultural Heritage and History of Science & Technology, University of Science and Technology Beijing, Beijing 100083, China; d202310767@xs.ustb.edu.cn (M.W.); zhangzhiyue98@163.com (Z.Z.); hanliuyang@ustb.edu.cn (L.H.); 2 Baotou Museum, Baotou 014010, China; mule13948421468@126.com

**Keywords:** paper relics, organosilane, reinforcement, preservation

## Abstract

Tetraethoxysilane (TEOS) is the most commonly used silicon-based reinforcement agent for conserving art relics due to its cost-effectiveness and commercial maturity. However, the resulting silica gel phase is prone to developing cracks as the gel shrinks during the sol–gel process, potentially causing severe damage to the objects being treated. In this study, dodecyltrimethoxysilane (DTMS) was introduced into TEOS to minimize this shrinkage by adding elastic long chains to weaken the capillary forces. The gel formed from the DTMS/TEOS hybrid material was transparent and crack-free, featuring a dense microstructure without mesopores or micropores. It exhibited excellent thermal stability, with a glass transition temperature of up to 109.64 °C. Evaluation experiments were conducted on artificially aged, handmade bamboo paper. The TEOS-based hybrid material effectively combined with the paper fibers through the sol–gel process, polymerizing into a network structure that enveloped the paper surface or penetrated between the fibers. The surface of the treated paper displayed excellent hydrophobic properties, with no significant changes in appearance, color, or air permeability. The mechanical properties of the treated bamboo paper improved significantly, with longitudinal and transverse tensile strengths increasing by up to 36.63% and 44.25%, respectively. These research findings demonstrate the promising potential for the application of DTMS/TEOS hybrid materials in reinforcing paper relics.

## 1. Introduction

Organosilane materials have shown potential as ideal paper relic preservation materials due to their dual advantages of both organic and inorganic properties. Characterized by Si–O–Si bonds as the primary skeleton, these materials possess thermal and chemical stability far superior to general polymer materials, resembling the properties of inorganic materials. During the sol–gel process, organosilane will react with the hydroxyl groups upon cellulose condensation, forming Si–O–C bonds that can effectively reinforce the paper. Furthermore, organosilane materials contain Si–C bonds with diverse organic groups, enabling the introduction of various groups to achieve deacidification [1], hydrophobicity [2], antimicrobial [3], and other desired effects.

Currently, silane coupling agents have been involved in the widespread practice of paper heritage protection. These agents belong to a class of reactive silanes that possess organic functional groups. Some of these compounds carry organic groups, such as alkyl, vinyl, amino, epoxy, or sulfhydryl groups, which enhance reactivity with organic compounds and introduce various functionalities into the resulting polymers. However, some silanes contain inorganic groups, such as alkoxy, aryloxy, acyl, and chlorine, which have excellent reactivity with the surfaces of inorganic substances [4,5,6]. When applied to paper, silane will undergo hydrolysis to produce silanols. These silanols will then dehydrate, condense, and crosslink to form oligosiloxanes, and the silanol groups present in oligosiloxanes will adhere to the paper’s surface [7]. Using the silane coupling method enables the grafting and modification of paper cellulose [8], introducing various functional groups into the paper and thereby endowing it with a wide range of appealing properties. It has been reported that silane coupling agents could bestow surface hydrophobicity and water resistance upon the paper [9,10] and even transform it into a catalyst medium or a drug carrier [11,12,13].

The application of silane coupling agents has also been explored in the preservation of paper relics. Chen et al. [14] and Shen et al. [15] both employed three silane compounds, namely, N-aminoethyl-3-aminopropyl-triethoxysilane (AETAPTES, trademarked as KH791), 3-aminopropyl-diethoxymethylsilane (AMDES, trademarked as JH-M902), and 3-aminopropyl-triethoxysilane (APTES, trademarked as KH550), for the reinforcement treatment of Xuan paper. These agents were dissolved in anhydrous ethanol to form a reinforcing solution. When the paper was immersed in this solution, the studies found that all three silanes effectively reinforced the paper, enhancing its tensile strength and folding durability. Among these, AETAPTES demonstrated the most notable reinforcement effect. Specifically, when applied at a concentration of 15%, the solutions increased the tensile strength of the paper by approximately threefold, and the folding endurance was 60 times greater.

Since 2006, researchers from the Muséum National d’Histoire Naturelle in France have conducted a comprehensive series of studies focusing primarily on the utilization of amino-functionalized silanes for paper conservation [1,16,17,18,19,20]. For the main purpose of deacidification, these studies evaluated various silanes, including 3-aminopropyltrimethoxysilane (ATMS), 3-aminopropyltriethoxysilane (ATES, identical to APTES), 4-amino-3,3-dimethylbutyltrimethoxysilane (ADBTMS), hexamethyl disiloxane (HMDS), and dimethylaminopropylmethyl diethoxysilane (DMAPDES), as well as other silane coupling agents, along with their corresponding compounds. These agents were tested for their efficacy in protecting both naturally aged and artificially aged paper. The results revealed that these silane coupling agents, apart from their deacidifying capabilities, could significantly enhance the mechanical properties of paper, such as tensile strength and folding endurance, by forming a robust network structure on the fiber surfaces.

Current studies have established that organosilane materials exhibit favorable reinforcing effects on paper relics; however, most of the silane coupling agents used either are costly or rely on laboratory synthesis. Considering the need for a substantial quantity of materials to support the preservation of paper relics, it remains imperative to explore cost-effective and readily available alternatives. Tetraethoxysilane (TEOS), a commercially viable and economical organosilane material, is most frequently utilized in heritage conservation [21] and offers a promising avenue if modifications can be made to TEOS to align with the performance requirements of paper relic protection.

The primary challenge with TEOS is its susceptibility to cracking upon gelation. This cracking can be attributed to two primary causes, namely, capillary stress during gel drying and the volatilization of water and alcohols during condensation [22]. Since the cracking of organosilanes may compromise the integrity of the artifact matrix, it has become imperative to engineer these materials with anti-cracking modifications. Although numerous strategies exist to address this issue, two primary methods remain particularly relevant for artifact preservation. First, the introduction of elastic chain segments may enhance the flexibility of the material, thereby resisting capillary stress during shrinkage [23]. For example, the incorporation of polydimethylsiloxane (PDMS) can densify the silicone gel structure [24,25,26]. In addition, the utilization of nanoparticles can increase the elastic modulus and gel pore size of the protective material, effectively mitigating capillary stress. This approach has been demonstrated through the use of nanoparticles such as TiO_2_, SiO_2_, and A1_2_O_3_ [27,28,29]. Furthermore, both methods can be combined to modify organosilanes. For example, Son et al. introduced nanometer-sized (3-glycidoxypropyl) trimethoxysilane (GPTMS) and polyhedral oligomeric silsesquioxane (POSS) with elastic chain segments into TEOS, resulting in a hybrid material providing a crack-free gel [30].

This study utilized inexpensive and widely available ethyl orthosilicate (TEOS) as the baseline material. To enhance its properties, dodecyltrimethoxysilane (DTMS) with flexible long chains was integrated into the TEOS agent, which resulted in a series of formations of DTMS/TEOS hybrid material agents through a compounding process. These DTMS/TEOS agents were evaluated based on their curing performance and were subsequently utilized to protect and treat aged bamboo paper. The color difference, mechanical properties, air penetration, and microstructure of the treated aged paper were characterized to assess the overall effectiveness of the DTMS/TEOS hybrid material for reinforcement and preservation.

## 2. Results and Discussion

### 2.1. DTMS/TEOS Hybrid Material

#### 2.1.1. Curing Performance

In this study, we prepared a series of hybrid materials with varying mass ratios of MTDS and TEOS (W_DTMS_/W_TEOS_ = 1:9, 2:8, 3:7, 4:6, and 5:5) and compared their curing effects. These five groups of materials were denoted as DT1 to DT5, respectively.

The appearance of the hybrid materials typically serves as a perceptive and fundamental criterion for assessing their curing performance. To ensure good curing and drying without cracking, the hybrid materials would need to be colorless and transparent to preserve the original appearance of the heritage samples during paper sample treatment. In addition, organosilanes undergo volume contraction during the sol–gel process, and excessive contraction can damage the paper fiber structure. Therefore, smaller volumetric shrinkage of the hybrid materials was sought.

**Figure 1:**
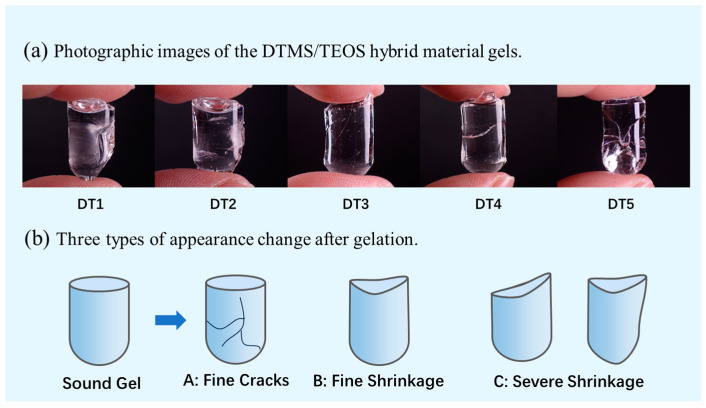
Appearance evaluation of the DTMS/TEOS hybrid material gels. (**a**) Photographic images of the DTMS/TEOS hybrid material gels; (**b**) three types of appearance change after gelation.

Images of the DTMS/TEOS gels are shown in Figure 1a. As presented in Figure 1b, the appearance changes of the hybrid materials after gelation could be described as three typical cases. In this study, the cracks generated in the gels of all groups were confirmed as small cracks that did not cause the gels to become fragile, namely, fine cracks. The volume shrinkage of each formulation after gelation was different. For some groups, we observed that the shrinkage of the gel mainly occurred on the oxygen contact surface, and no significant impact was observed on the overall size and shape, which was defined as fine shrinkage. However, some gels with different formulations showed irregular shrinkage, which seriously affected the curing effect and was defined as severe shrinkage.

Table 1 presents information on the types of changes in appearance after gelation of each group, according to Figure 1b, as well as their volumetric shrinkage rates, mass retention rates, and curing times. The results indicated that under the catalytic conditions of 0.2 wt.% concentration dibutyltin dilaurate (DBTL), the DTMS/TEOS hybrid materials could meet the required curing performance. When the mass fraction of DTMS was only 10%, the system could produce a completely transparent gel with only fine cracks, and the volumetric shrinkage varied little among the five groups, mostly falling between 40% and 50%. When the mass fraction of DTMS was ≥40%, the gels exhibited low volumetric shrinkage, making them less prone to severe shrinkage and cracking. Among these, the DT5 agent (W_DTMS_/W_TEOS_ = 5:5) demonstrated the most prominent anti-shrinkage effects, with the volumetric shrinkage of the gels reaching the lowest average value of only 36.72%.

During the sol–gel process, organosilanes will release a significant amount of water and alcohol, with some organosilanes possessing a certain degree of volatility. Therefore, it was necessary to assess the retention of active ingredients by examining the mass retention rate of the hybrid materials. A higher retention rate typically indicates a greater coating effect on the paper. We found that the mass ratio of DTMS and TEOS had no significant impact on the mass retention rate of the DTMS/TEOS hybrid materials and generally remained within the range of 55–65%. Notably, the mass retention rate reached its highest point of 63.23% when W_DTMS_/W_TEOS_ was set to 5:5.

Curing time also served as a crucial factor in determining whether the hybrid material agent could meet the required properties for practical applications. A longer curing time tended to enhance the stability of formulations during long-term practical applications, providing ample time for large-scale construction and fine processing. When the DTMS mass fraction increased, the full curing time also increased. The DT5 group, with the highest DTMS concentration, required the longest curing time at approximately 307 h. Taking both the curing time and cost into consideration, a formulation with a W_DTMS_/W_TEOS_ ratio of 5:5 emerged as the most preferred option for maximizing active ingredient retention.

#### 2.1.2. Thermal Stability

To investigate the thermal stability of the DTMS/TEOS hybrid materials, two groups with the largest and lowest W_DTMS_/W_TEOS_ ratios (W_DTMS_/W_TEOS_ = 1:9 and 5:5) were selected for DSC testing. The glass transition temperatures (Tg) of the hybrid materials were determined based on the DSC curves.

**Figure 2:**
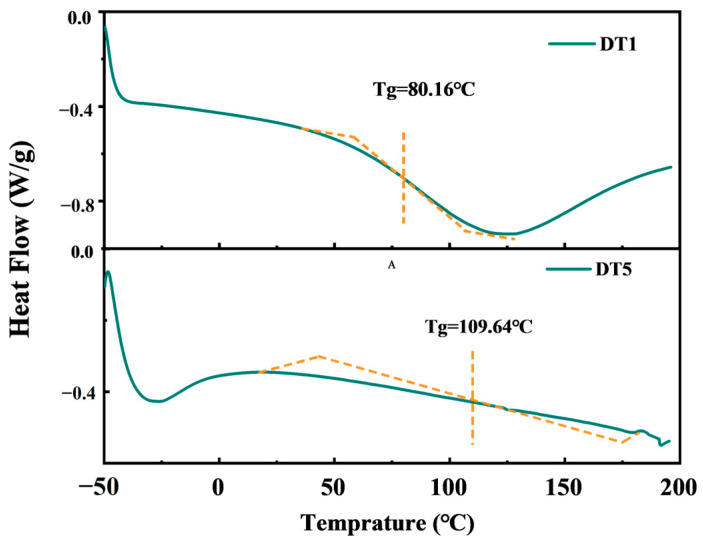
DSC curves of the DTMS/TEOS hybrid materials.

As shown in Figure 2, for the DTMS/TEOS hybrid materials, the Tg values were 80.16 °C and 109.64 °C when W_DTMS_/W_TEOS_ = 1:9 and 5:5, respectively. Notably, all of the Tg temperatures were significantly higher than the temperature of a typical use environment (room temperature). These results indicated that the prepared hybrid materials would remain in a glassy state at the use environment temperature. In this state, the molecular chains and chain segment motions were effectively frozen, with only the atoms constituting molecular vibrations at their equilibrium positions. Consequently, the hybrid materials exhibited superior thermal stability performance [31], and due to their high Tg temperatures, these hybrid materials possessed greater rigidity.

#### 2.1.3. Micromorphology and Porosity

**Figure 3:**
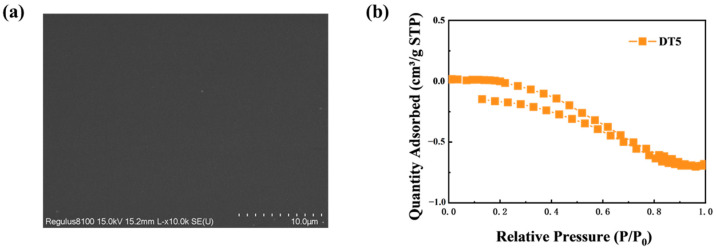
(**a**) SEM image under 10,000× magnification and (**b**) N2 adsorption–desorption curve of the TEOS/DTMS hybrid material.

The specific surface and porosity of the optimally formulated hybrid material were analyzed. The specific surface area of the DT5 agent was found to be −0.0699 m^2^/g. When combined with the N_2_ adsorption–desorption graphs (Figure 3b), we observed that within the tested range, the hybrid material gel did not contain micropores or mesopores.

The microstructure of the DT5 gel was observed under a scanning electron microscope. As revealed in Figure 3a, the surface of the polymer exhibited a flat, wrinkle-free, crack-free, dense, and uniform plate-like structure under 10,000× magnification. These characteristics indicated that the hybrid material lacked corresponding pores, which aligned with the porosity test results. We observed that DTMS mitigated the propensity of TEOS-based gel to cracking, and this was achieved, in part, by introducing flexible chains that could withstand capillary stress during the shrinkage process.

This dense and non-porous hybrid material gel, if uniformly encapsulated on the paper surface or grown in the pores formed by the fibers, could block the invasion of most external pollutants into the paper, such as dust, vermin, and mold [32,33].

### 2.2. Effectiveness of DTMS/TEOS Hybrid Materials for the Reinforcement of Aged Bamboo Paper

#### 2.2.1. Appearance and Surface Hydrophobicity

**Figure 4:**
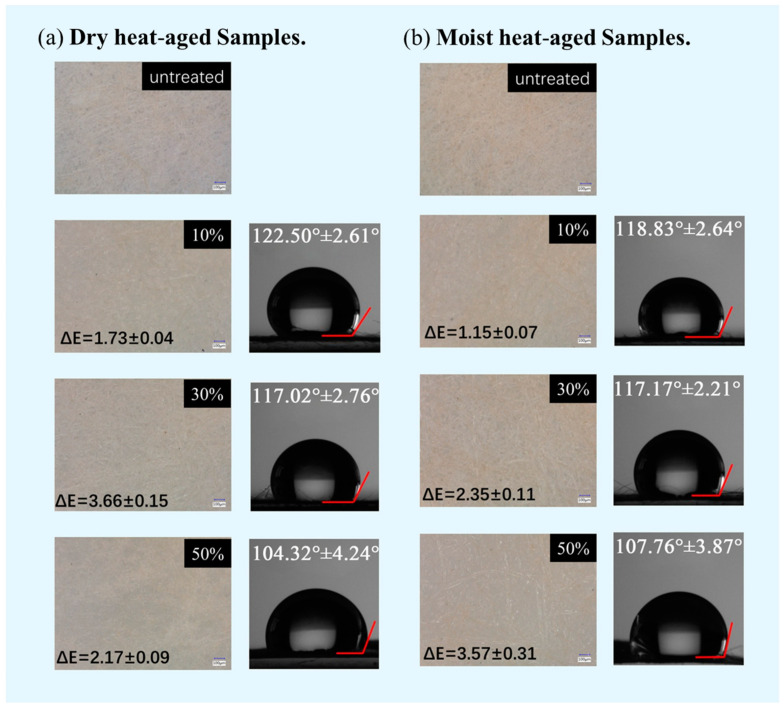
Changes in color and surface properties of the aged paper samples before and after treatment with DT5 agents in different concentrations. (**a**) Dry heat-aged samples; (**b**) moist heat-aged samples.

As shown in Figure 4, the color change in DT5 at the three concentrations (10%, 30%, and 50%) tested on aged bamboo paper was generally indistinct, according to the color difference assessment level [34]. In particular, at a low concentration of 10%, DT5 hardly alters the color of the paper. We observed that as the concentration of the agent increased, more produced polymer coated the fiber surface and filled the pores, leading to a noticeable color difference in the paper. When the concentration of DT5 reached 50%, the paper demonstrated a distinctive, transparent texture. This was because the initial roughness of the paper surface caused light to scatter, resulting in a very low visual gloss on the surface. However, with the deposition of 50% DT5, the depressions between the fibers were filled. As a result, the smoothness of the paper surface and its gloss were significantly enhanced. Therefore, even though a high concentration of DT5 may better fill the pores and cracks on the paper surface, it is not recommended to exceed a usage concentration of 30% in order to avoid potential significant detrimental effects on the appearance of the paper relics.

Cellulose, rich in hydroxyl groups, is highly hydrophilic and prone to absorbing moisture. Therefore, paper may over-absorb water in high environmental humidity conditions, leading to cellulose deliquescence and a decrease in the strength of its structure and organization. In addition, high humidity can facilitate the atmospheric deposition of hazardous dust and gases soluble in water, potentially corroding the paper. Furthermore, high humidity can promote the growth and reproduction of microorganisms, making it easy for mold to grow on paper [35]. To mitigate issues such as water stains, filth, adhesion, and wrinkles, it was essential that the protected paper exhibit hydrophobic properties that could block environmental water erosion.

Contact angle can serve as an indicator of paper surface hydrophobicity after treatment with a protective agent. When the contact angle decreased, the wettability of the solid surface improved and became more hydrophilic. Conversely, a larger contact angle indicated a more hydrophobic surface, signifying superior waterproof performance. We captured images of the contact angle on the surface of the paper after 10 s of exposure to water. Both the untreated sound bamboo paper and the two groups of aged paper lacked hydrophobicity, with water droplets being absorbed almost instantly; hence, no contact angle photos were obtained. In contrast, the treated aged bamboo paper consistently maintained pronounced hydrophobicity. As shown in Figure 4, the three concentrations of DT5 tested on aged bamboo paper had the ability to transform a fully wetted surface (contact angle of θ = 0°) into a non-wetted surface (contact angle of θ > 90°). This change highlighted the effectiveness of DTMS/TEOS hybrid materials in imparting hydrophobicity to aged bamboo paper. In summary, a 10% concentration of DT5 was sufficient to provide a hydrophobic surface for aged bamboo paper without altering its appearance.

#### 2.2.2. Tensile Strength

The resistance of paper to aging will significantly impact its service life, making it crucial to investigate its aging resistance, with mechanical properties serving as key evaluation indicators. The most commonly used metric for assessing the mechanical properties of paper is tensile strength [36,37,38]. Therefore, to enhance the aging resistance of paper, the tensile strength of paper treated with a protective agent should be increased to a certain extent.

**Figure 5:**
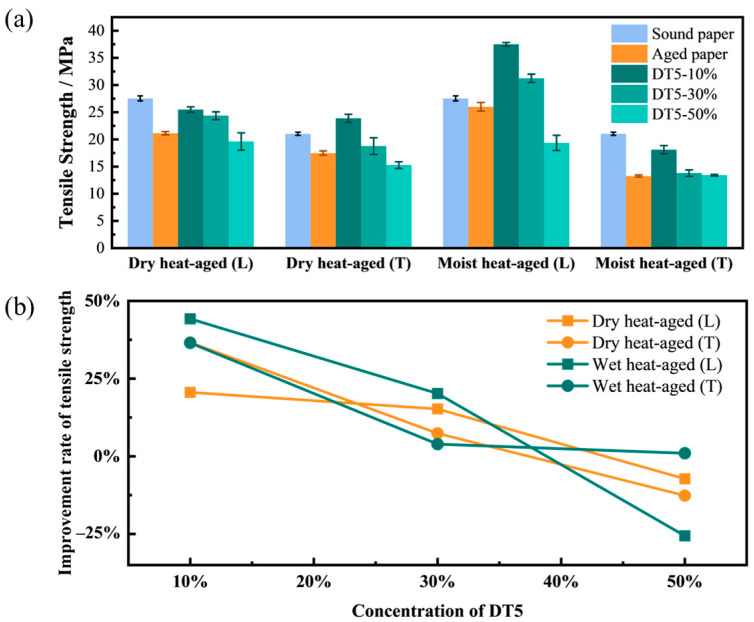
(**a**) Mean value and standard deviation of the tensile strength, and (**b**) change rate of the tensile strength of aged bamboo paper before and after treatment with DT5 agents at different concentrations, where T denotes the transverse direction and L signifies the longitudinal direction.

The mean and standard deviation of the tensile strength of aged bamboo paper before and after DT5 agent treatment are presented in Figure 5a, while the rate of change in tensile strength is shown in Figure 5b. The dry heat-aged bamboo paper exhibited longitudinal and transverse tensile strengths of 21.13 ± 0.31 MPa and 17.47 ± 0.39 MPa, respectively. Conversely, the moist heat-aged bamboo paper showed longitudinal and transverse tensile strengths of 25.99 ± 0.79 MPa and 13.26 ± 0.19 MPa, respectively. Notably, when the concentration of DT5 was 10%, the most significant enhancement effect was observed. In the dry heat-aged bamboo paper, the longitudinal and transverse tensile strengths were 25.48 ± 0.50 MPa and 24.87 ± 0.75 MPa, increasing by 20.59% and 36.63%, respectively. Similarly, in the moist heat-aged bamboo paper, the tensile strength values were 37.49 ± 0.35 MPa and 18.10 ± 0.76 MPa, increasing by 44.25% and 36.50%, respectively. The untreated sound paper demonstrated a longitudinal tensile strength of 27.54 ± 0.46 MPa and a transverse tensile strength of 21.03 ± 0.29 MPa. It is evident that 10% DT5 was sufficient to restore the strength of aged bamboo paper to a level comparable to or even higher than that of sound bamboo paper. However, when the concentration of DT5 increased, the increase in tensile strength values for both types of aged bamboo paper decreased in both directions. At a DT5 concentration of 50%, the tensile strength even dropped below the initial value. This finding aligns with the previous conclusion that 10% DT5 exhibited the most favorable enhancement effect, while higher concentrations of DT5 were counterproductive for the preservation of paper artifacts.

#### 2.2.3. Microstructure

**Figure 6:**
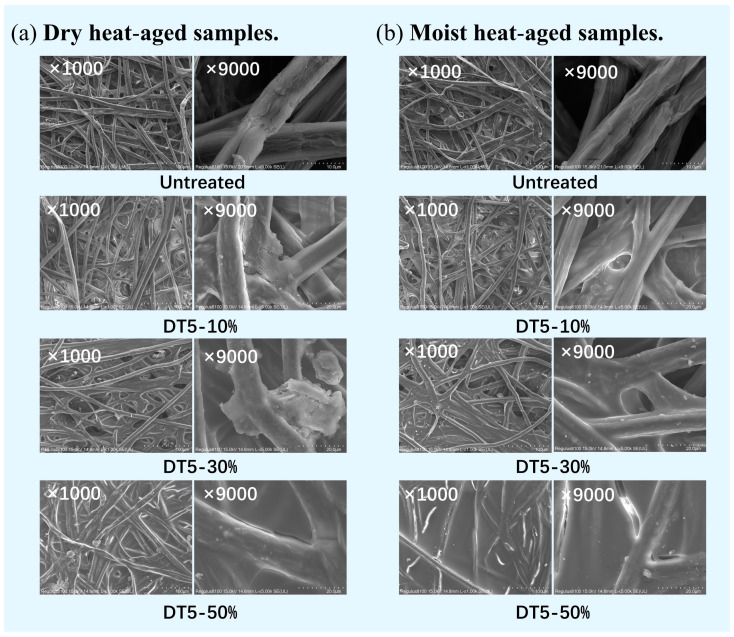
Microstructure of the aged bamboo paper before and after treatment with DT5 agents at different concentrations. (**a**) Dry heat-aged samples; (**b**) moist heat-aged samples.

Scanning electron microscopy (SEM) analysis of the paper samples was conducted to observe the distribution of the hybrid materials, as illustrated in Figure 6. Bamboo paper primarily consists of slender fibers intertwined, along with a few flat and wide fibers. Prior to the protection treatment, the bamboo paper fibers exhibited cracks, and the interlacing connections between fibers were loose, with some of the interlacing connected by membranes, presumably due to the viscous additives used in the papermaking process [15]. After treatment with DT5 agents, the fibers were uniformly coated with the protective agent, resulting in a smoother surface. Block-like and membrane-like polymers formed at the intersections of the fibers, resulting in a more intimate connection between the fibers. This observation at the microscale might explain the reason behind the enhanced tensile strength of aged bamboo paper when treated with 10% and 30% DT5.

However, when the concentration of DT5 reached 50%, the fibers were almost completely covered by the hybrid material, and the fiber pores were basically non-existent. We speculated that a high concentration of agents would lead to the formation of excessive hybrid material polymers inside the paper or even on the surface of the paper. The produced silicone polymer became the dominant component of the paper after curing; however, it did not have the interwoven fiber microstructures of sound paper, which provided high flexibility. Furthermore, when the agent concentration was excessively high, it could not only distribute within the gaps but also extensively infiltrate into the fibers, resulting in fiber damage during the curing process. As a result, the excessive polymers made paper into a resin-like film and changed the appearance as well as the mechanical properties of the paper.

#### 2.2.4. Water Vapor Transmission Rate

**Figure 7:**
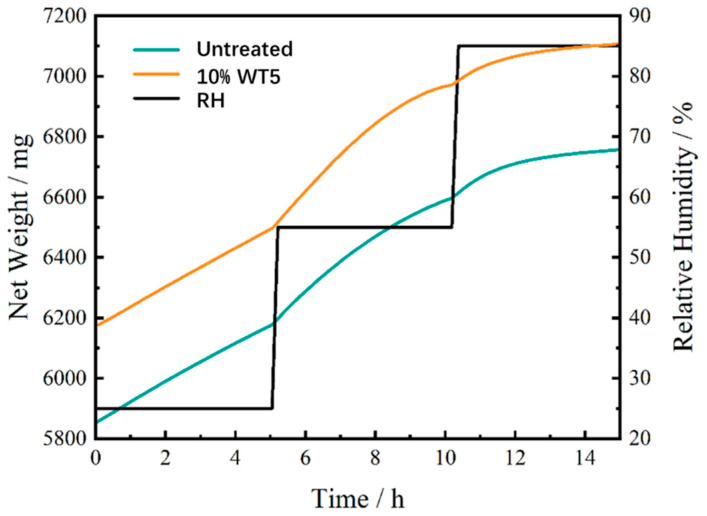
Mass–time curve of the paper sample before and after treatment with 10% DT5 agent.

The mass–time curves of the aged paper before and after treatment with 10% DT5 agent, obtained from the air permeability test, are shown in Figure 7. At relative humidity (RH) values below 25%, the curve was linear, allowing for the accurate computation of the water vapor transmission rate (WVTR). When the RH increased to 55%, the linearity of the curve persisted in the initial half, allowing for WVTR calculations. However, the second half of the curve started to approach a state of equilibrium, indicating that the molecular sieve reached saturation and ceased to absorb water. Similarly, at an RH value of 85%, the equilibrium portion of the curve could not be utilized for WVTR calculations due to the same saturation phenomenon.

At 25% RH, the linear mass change between 1.9 and 3.7 h was selected to calculate the WVTR, and at 55% RH, the corresponding interval between 5.4 and 6.1 h was chosen. The results of these calculations are summarized in Table 2, which indicates that regardless of treatment, the aged paper samples exhibited a high water vapor transmission rate. Notably, the DT5 agent had no significant impact on the air permeability of the paper.

For immovable paper artifacts such as historical decorative wall coverings and paper-based murals, they are significantly influenced by moisture migration from the walls to which they were adhered. As the walls absorb water from the ground, they may create a highly humid environment on the backside of the attached paper and cause it to become damp. A high WVTR facilitates the release of moisture from the wall through the paper into the open environment rather than allowing it to accumulate or be absorbed into the fibers, leading to mold formation. Hence, while 10% DT5 did partially fill the pore structure of the paper, it did not transform it into a completely sealed polymer film. The treated paper still allows moisture to pass through, thereby preventing excessive moisture-related risks.

## 3. Materials and Methods

### 3.1. Preparation of the Hybrid Materials

The organosilanes employed in this study consisted of tetraethoxysilane (Aladdin, Shanghai, China, 99.99%) and dodecyltrimethoxysilane (Aladdin, Shanghai, China, 93%). DBTL (Aladdin, Shanghai, China, 95%) was used as a catalyzer, and absolute ethanol (Aladdin, Shanghai, China, AR) was used as the solvent.

**Figure 8:**
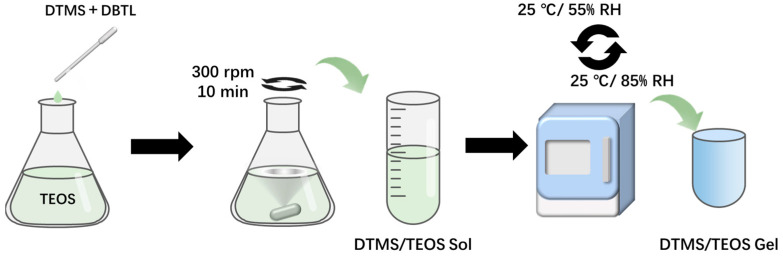
Diagram of DTMS/TEOS hybrid material agent preparation.

The DTMS/TEOS hybrid material agents were prepared according to the formulations provided in Table 3 and thoroughly mixed to obtain a homogeneous mixture. Then, 2 mL of the prepared DTMS/TEOS sol with 0.2 wt% of DBTL was added to a plastic centrifuge tube, weighed, and placed inside the temperature and humidity environmental test chamber (GSH-64, ESPEC, Osaka, Japan). The conditions were set as follows: 25 °C and 55% RH for 3 h, followed by 25 °C and 85% RH for another 3 h. This cycle was repeated every 6 h. The moisture curing process was carried out under these conditions until the mass of the material stopped changing, indicating that the reaction had finished. The resulting gel was removed from the centrifuge tube and observed as transparent and free of cracks; then, its mass retention and volume shrinkage were calculated. The entire preparation process is illustrated in Figure 8.

### 3.2. Paper Aging

The handmade bamboo paper for artificial aging, which is widely used in calligraphy and painting creation, restoration of ancient books, and other paper relics, was produced by Fuyang Yiguzhai Yuanshu Paper Co., Ltd. (Fuyang, Zhejiang, China). The basis weight of the paper is 19.0 g·cm^−2^, and the thickness is 0.03 mm. The bamboo paper was artificially aged by two aging methods, namely, dry heat aging and moist heat aging. The dry heat aging method was conducted according to the Chinese GB/T 464-2008 national standard [39]. The bamboo paper samples were subjected to dry heat aging in an electric constant-temperature blast-drying oven (DHG-9053A, Jinghong, China) under the following dry heat aging conditions: 105 °C and 24 days.

The moist heat aging method was conducted according to GB/T 22894-2008 [40]. The bamboo paper samples were subjected to moist heat aging in an environmental test chamber (SH-222, ESPEC, Osaka, Japan) under the following moist heat aging conditions: 80 °C, 65% RH, and 24 days.

### 3.3. Reinforcement of Paper Samples

**Figure 9:**
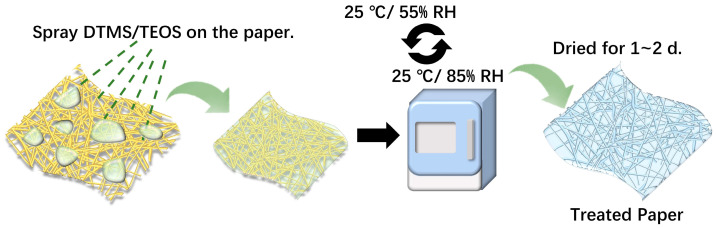
Diagram of the aged paper reinforcement process using DTMS/TEOS hybrid materials.

The prepared protective solution was diluted with ethanol at various concentrations (V_solution_:V_ethanol_ = 1:9, 3:7, and 5:5) and then thoroughly mixed. The resulting agent was then evenly sprayed onto the aged bamboo paper using spray bottles. To process 0.1 m^2^ of paper, approximately 1 milliliter of solution was used. Afterward, the treated paper was placed in an environmental test chamber (SH-222, ESPEC, Osaka, Japan) for moisture curing. The curing conditions were as follows: 25 °C and 55% RH for 3 h, followed by 25 °C and 85% RH for another 3 h. This cycle was then repeated every 6 h. The paper was left to dry for 1–2 days, and the moisture curing process was considered complete. The treating process is illustrated in Figure 9.

### 3.4. Specific Surface Area and Porosity

The pore structure of the materials was characterized by N_2_ adsorption–desorption analysis. In this experiment, N_2_ adsorption–desorption curves of DTMS/TEOS gels were obtained at 77 K using a specific surface and porosity analyzer (ASAP2460, Micromeritics, Norcross, Georgia, USA). The specific surface area of the samples was calculated based on the Brunauer–Emmet–Teller (BET) model, while the pore volume and average pore size of the samples were determined by analyzing the desorption data using the Barret–Joyner–Halenda (BJH) method.

### 3.5. Glass Transition Temperature

The glass transition temperature (Tg) can be defined as the point at which a material transitions from a glassy state to a highly elastic state. Below this temperature, the material will remain in a glassy state, with its internal molecular chains and chain segment movements effectively frozen. Once the temperature surpasses Tg, the material will enter a highly elastic state [31]. In practical applications, Tg can be used to determine the suitable temperature range for using the material. By assessing the Tg of the DTMS/TEOS gel, the thermal stability of the material within the desired use environment could be determined. For this experiment, a differential scanning calorimeter (DSC25, TA Instruments, New Castle, DE, USA) was used to analyze the Tg of the DTMS/TEOS hybrid materials. This method relied on detecting changes in the heat capacity before and after the glass transition to determine the magnitude of Tg, following the guidelines outlined in the GB/T 19466.2-2004 Plastics Differential Scanning Calorimetry (DSC) standard (Part 2: Determination of Glass Transition Temperature) [41].

### 3.6. Color Difference

The color difference (ΔE*) between the aged paper samples before and after treatment with the DT5 agents was measured by a spectrophotometer (CM-26D, Konica Minolta, Japan) under a D65 light source. For each sample, the color difference was determined based on five points. The following equation was used to calculate the color difference ΔE* for each point, and the average ΔE* value was calculated to represent the ΔE* of the entire sample [34]:

ΔE* = [(ΔL*)^2^ + (Δa*)^2^ + (Δb*)^2^]^½^, (1)

where L* ranges from 0 to 100 and indicates the change in color from black (dark) to white (light), a* varies from negative to positive and represents the change in color from green to red, and b* ranges from negative to positive and indicates the change in color from blue to yellow.

### 3.7. Contact Angle

In this experiment, a contact angle meter (JC2000DM, Zhongchen, China) was used to determine the contact angle and assess the hydrophobicity of the protected paper surface. Deionized water droplets were applied to the treated paper samples, and a fixed image was captured after 10 s. Three measurement points were taken for each sample, with a five-point fitting method used to measure the contact angle value at each point. The average of the three points was then calculated to determine the contact angle value of the sample.

### 3.8. Tensile Test

Tensile tests were conducted on the paper samples to assess their mechanical properties, and a thermomechanical analyzer (TMA7100, HITACHI, Tokyo, Japan) was utilized in this study. The TMA method enabled the acquisition of highly reproducible tensile strength data from millimeter-sized samples, making it particularly suitable for micro-destructive mechanical testing of fragile organic cultural relics [42]. Further details regarding this method can be found in our previous research paper [43]. The paper samples were cut into rectangular specimens measuring 15 mm × 2 mm in both the longitudinal and cross directions using a paper cutter. The thermomechanical analyzer (TMA) was equipped with a metal tensile attachment to conduct the tensile tests, where the test length, or fixture spacing (*L*), was set to 10 mm. The specimens were held at both ends using the fixtures, with the sample tubes and tensile probes firmly fixed in place. The tests were conducted in a laboratory room environment (25 ± 1 °C, 45% ± 3% RH) after the paper sample was conditioned in this test environment for more than 48 h with an initial load of 10 mN and a loading rate of 250 mN/min. The probe displacement and load were recorded over time until the specimen broke, and the stress (*σ_f_*) and strain (*ε_f_*) were calculated using Equations (2) and (3), respectively, to generate the stress–strain curve of the specimen:

(2) $$\sigma_{f}=\frac{F}{bd},$$

(3) $$\varepsilon_{f}=\frac{\Delta L}{L},$$

where *σ_f_* is the fracture stress (MPa), *F* is the real-time load recorded by TMA (N), *b* is the width of the fracture surface of the specimen (mm), *d* is the thickness of the fracture surface of the specimen (mm), *ε_f_* is the fracture strain, Δ*L* is the real-time displacement recorded by TMA (mm), and *L* is the specimen test length (mm), i.e., the fixture spacing.

The maximum stress at the time of fracture was recorded as the tensile strength *σ*, *b* was measured using a three-dimensional video microscope (VHX-6000, KEYENCE, Osaka, Japan) with an accuracy of 0.01 mm, and *d* was measured using a digital micrometer with an accuracy of 0.001 mm.

### 3.9. Microstructure

A scanning electron microscope (SEM) was used to examine the microstructure of the paper and DTMS/TEOS hybrid material gels. This analysis was used to assess fiber breakage in the paper, the pore structure and cracking of the hybrid material gels, and the distribution of the hybrid material polymers within the paper fibers. In this experiment, an ultra-high-resolution field-emission scanning electron microscope (Regulus 8100, HITACHI, Tokyo, Japan) was used. The surface of the paper samples was subjected to a gold spraying treatment. The microscope was operated with an accelerating voltage of 15 kV, a resolution of 0.7 nm, a working distance of 15 mm, and a secondary electron imaging (SE) working mode.

### 3.10. Water Vapor Transmission Test

A high-throughput dynamic moisture-adsorption tester (SPSx-1μ, ProUmid, Ulm, Germany) was utilized to assess the permeability of the samples under identical temperature conditions and various relative humidity levels. In the test setup, approximately 5 g of molecular sieve was placed in the sample tray. The bamboo paper was cut into a circular shape (radius = 4 cm) and securely attached to the fixture on the sample tray. The test parameters were set as follows: test range: 25–85% RH; humidity gradient: 30% RH; temperature: 25 °C. The change in the net weight of the sample over a specific period was determined, and the water vapor transmission rate (*WVTR*) of the bamboo paper was calculated using the following equation:

(4) $$WVTR=\frac{W_{\mathrm{end}}-W_{\mathrm{start}}}{\pi R^{2}\cdot T}$$

where *W*_start_ is the net weight of the paper sample at the selected start time (mg), *W*_end_ is the net weight at the selected end time (mg), *R* is the radius of the bamboo paper sample (which was 4 cm in this study), and *T* is the total time from the selected start time to the end time (h).

## 4. Conclusions

This study demonstrated that the DTMS/TEOS hybrid materials possessed excellent anti-cracking properties. The hybrid materials provided transparent and dense gels with no severe cracks. We observed that with the increase in DTMS addition, the resulting gels were less prone to cracking. In addition, these gels exhibited good thermal stability, which was attributed to the flexible chains introduced by DTMS, and this helped to resist capillary stress during shrinkage. The experimental results indicated that under the catalytic conditions of a 0.2% mass concentration of DBTL, the DTMS/TEOS hybrid materials achieved good curing effects across various mass fraction ratios. The DT5 agent (with a W_DTMS_/W_TEOS_ = 5:5) achieved the highest mass retention rate of 63.23%. At this ratio, the volume shrinkage was minimal at 36.72%, and the complete curing time was 307 h, making it the most preferred formulation.

In addition, we observed that a 10–50% concentration of DT5 agent in the ethanol solution effectively reinforced and protected aged paper. Notably, there was no significant change in the color appearance of the treated paper, and its surface exhibited strong hydrophobicity. Among the various tested concentrations, the 10% concentration exhibited the best reinforcement effect. After dry heat aging, the tensile strength of the bamboo paper increased by 20.59% and 36.63% in the longitudinal and cross directions, respectively. Similarly, the moist heat-aged paper samples resulted in 44.25% and 36.50% increases in tensile strength in the longitudinal and cross directions, respectively. The DTMS/TEOS hybrid material formed a strong and dense network structure on the surface and between the fibers of the paper, filling the pores and imparting strong hydrophobicity to the paper. This pore closure significantly enhanced the mechanical properties and hydrophobicity of the paper while maintaining its air permeability almost entirely.

In conclusion, the developed DTMS/TEOS hybrid material demonstrated a positive reinforcement and preservation effect on paper cultural relics. In addition, the material demonstrated ease of preparation, low cost, and wide accessibility, making it a promising choice for future research and applications for paper relic preservation.

## Figures and Tables

**Table 1:** Appearance changes, mass retention rates, volumetric shrinkage rates, and curing times of the DTMS/TEOS hybrid materials.

Group	Appearance Change	Mass Retention Rate	Volumetric Shrinkage Rate	Curing Time
DT1	C	57.06%	42.11%	210~213 h
DT2	A C	55.51%	48.23%	
DT3	A	54.18%	50.12%	261 h
DT4	A B	59.77%	40.43%	
DT5	A	63.23%	36.72%	307 h

**Table 2:** WVTR values of 10% DT5-treated aged paper at 25% and 55% RH determined by air permeability testing.

RH	Group	*W*_start_ (mg)	*W*_end_ (mg)	T (h)	WVTR [g/(m^2^·d)]
25%	Untreated	5982.087	6098.696	1.8	443.718
	10%DT5	6296.096	6413.247	1.8	445.780
55%	Untreated	6217.031	6292.713	0.7	740.528
	10%DT5	6541.522	6627.535	0.7	841.614

**Table 3:** Formulations of the DTMS/TEOS hybrid material agents.

Group	W_DTMS_/W_TEOS_
DT1	1:9
DT2	2:8
DT3	3:7
DT4	4:6
DT5	5:5

## Data Availability

The data presented in this study are available on request from the corresponding author.
